# Enhanced DNA-repair capacity and resistance to chemically induced carcinogenesis upon deletion of the phosphatase regulator NIPP1

**DOI:** 10.1038/s41389-020-0214-3

**Published:** 2020-03-02

**Authors:** Iris Verbinnen, Shannah Boens, Monica Ferreira, Kathelijne Szekér, Louise Van Wijk, Aleyde Van Eynde, Mathieu Bollen

**Affiliations:** Laboratory of Biosignaling & Therapeutics, KU Leuven Department of Cellular and Molecular Medicine, University of Leuven, Leuven, Belgium

**Keywords:** Cancer models, Cell biology

## Abstract

Nuclear Inhibitor of PP1 (NIPP1) is a conserved regulatory subunit of protein phosphatase PP1. The selective deletion of NIPP1 in mouse liver parenchymal cells or skin epidermal cells culminates in a late-onset hyperproliferation of a subset of resident progenitor cells. Although a hyperplastic phenotype is usually tumor promoting, we show here that the absence of NIPP1 conferred a strong resistance to chemically induced hepatocellular or skin carcinoma. The ablation of NIPP1 did not affect the metabolism of the administered mutagens (diethylnitrosamine or 7,12-dimethylbenz[a]anthracene), but reduced the conversion of mutagen-induced covalent DNA modifications into cancer-initiating mutations. This reduced sensitivity to mutagens correlated with an enhanced DNA-damage response and an augmented expression of rate-limiting DNA-repair proteins (MGMT in liver, XPD and XPG in skin), hinting at an increased DNA-repair capacity. Our data identify NIPP1 as a repressor of DNA repair and as a promising target for novel cancer prevention and treatment therapies.

## Introduction

NIPP1 (38 kDa), encoded by *PPP1R8*, is a ubiquitously expressed nuclear protein that targets phosphoproteins for regulated dephosphorylation by associated protein Ser/Thr phosphatase PP1^1–3^. Phosphoproteins are recruited by the phosphate-binding loop of the forkhead-associated (FHA) domain of NIPP1 and include a transcription factor (protein methyltransferase EZH2), two pre-mRNA splicing factors (SF3B1/SAP155 and CDC5L), and a DNA-replication factor (protein kinase MELK)^4–7^. At first glance, the FHA ligands of NIPP1 are functionally disconnected. However, recent data suggest that they all contribute to the cellular response elicited by DNA damage. Indeed, CDC5L is an activator of the DNA-damage checkpoint kinase ATR^8^, SF3B1 and MELK promote the expression of DNA-repair factors^9,10^, and EZH2 contributes to the resolution of stalled replication forks^11^. Interestingly, NIPP1 itself may also have a role in the DNA-damage signaling as expression of a fusion of PP1 and NIPP1 induced DNA damage, including double-strand breaks^3^. PP1-NIPP1 fusions with inactivating mutations of the PP1 moiety or FHA-domain were less effective in eliciting DNA damage, suggesting that NIPP1 opposes DNA repair through its control on the PP1-mediated dephosphorylation of FHA ligands.

The global deletion of NIPP1 in mice was embryonic lethal at the gastrulation stage^12^. Likewise, the postnatal ablation of NIPP1 in testis resulted in a complete loss of germ cells^13^. In contrast, the selective deletion of NIPP1 in mouse skin epidermal cells or liver parenchymal cells was less detrimental, the major phenotype being a slow-onset hyperproliferation of specific epidermal or biliary progenitor cells, respectively^14,15^. This enabled us to use the skin knockout (SKO) and liver knockout (LKO) models to further delineate the contribution of NIPP1 to the cellular DNA-damage response. For these studies, we provoked DNA damage, and ultimately tumor formation, using well-characterized chemicals. Skin carcinogenesis was induced in two stages, using consecutively the tumor-initiating mutagen 7,12-dimethylbenz[a]anthracene (DMBA) and the tumor-promoting phorbol ester 12-*O*-tetradecanoylphorbol-13-acetate (TPA)^16^. Hepatocellular carcinoma was provoked by a single injection of the DNA-alkylating agent diethylnitrosamine (DEN)^17^. Our data demonstrate that the NIPP1 SKOs and LKOs, despite the associated hyperproliferation phenotype, were strongly resistant to induced mutagenesis and carcinogenesis. This resistance correlated with an increased expression level of key DNA-repair factors. These findings further characterize NIPP1 as a repressor of the DNA-repair capacity and provide a rationale for the development of NIPP1 modulators in cancer prevention and treatment therapies.

## Results

### Resistance of *Ppp1r8*^*−/−*^ keratinocytes to DMBA/TPA-induced skin carcinogenesis

**Fig. 1:**
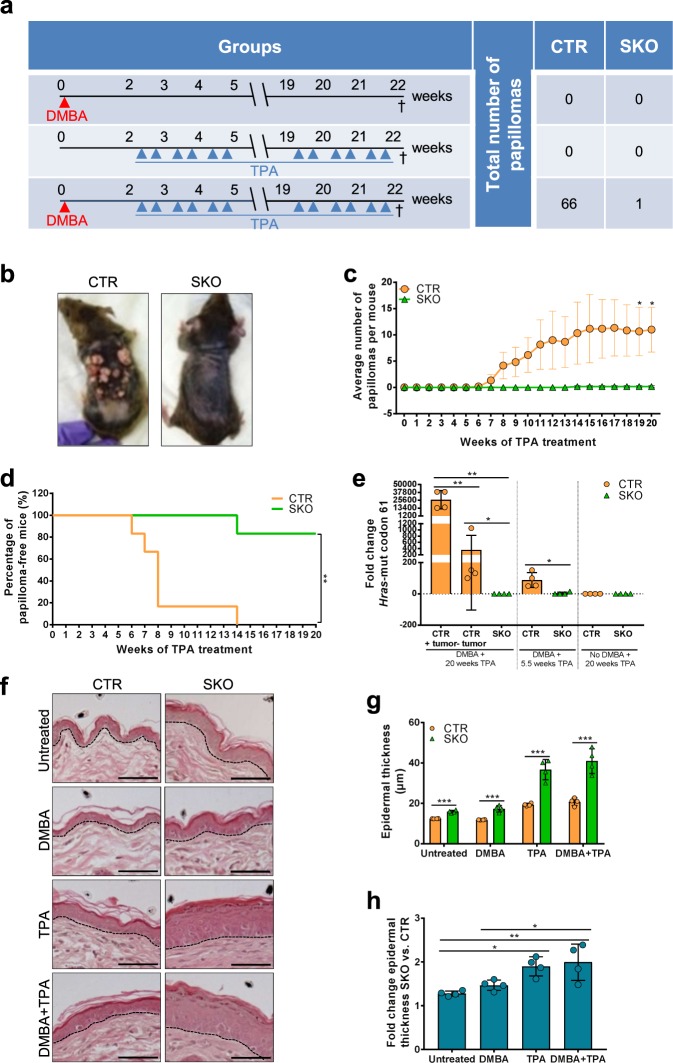
*Ppp1r8*^*−/−*^ keratinocytes are resistant to DMBA-induced mutagenesis. **a** Procedure for the application of DMBA, TPA or DMBA + TPA on the back skin of CTR and SKO mice. Also shown are the total number of papillomas (6 mice in each condition, except for the TPA condition: 5 CTR and 7 SKO) that were macroscopically detected at the time of sacrifice (†). **b** Representative picture of a CTR and SKO mouse at the end of the combined DMBA + TPA treatment. All mice that received this treatment are pictured in suppl. Fig. 1c. **c** Average number of papillomas formed in CTRs and SKOs during the combined DMBA + TPA treatment. Results are means ± SEM (*n* = 6). **d** Percentage of papilloma-free CTR and SKO mice during the combined DMBA + TPA treatment. **p < 0.01 in Log-rank test (*n* = 6). **e** DMBA-induc**e**d mutations of *Hras* at codon 61, as detected by qPCR in genomic DNA isolated from the back skin of CTR and SKO mice treated with TPA, DMBA + TPA (5.5 weeks), or DMBA + TPA (20 weeks). For CTR mice, tumors and tumor-free tissue were analyzed separately. *Actin* was used as a housekeeping gene for normalization. *p < 0.05; **p < 0.01 in unpaired Student’s *t*-test. The results are expressed as means ± SD for 4 mice in each condition. **f** H&E staining of the back skin of (un)treated CTR and SKO mice. The treatment with TPA was for 20 weeks. Scale bars, 50 µm. **g** Quantification of epidermal thickness in (un)treated CTRs and SKOs. Data are represented as means (*n* = 4) ± SD. ***p < 0.001 in unpaired Student’s *t*-test. **h** Fold change of epidermal thickness in SKOs versus CTRs. Data are shown as means ± SD (*n* = 4). *p < 0.05; **p < 0.01 in two-way ANOVA with Tukey’s multiple comparisons test.

In the NIPP1 skin knockout mice (SKOs) that we generated one *Ppp1r8* allele is disrupted in all somatic cells and the second, floxed *Ppp1r8* allele, was selectively inactivated in keratinocytes by CRE recombinase under control of the *Keratin-14* promoter^15^. Heterozygous *Ppp1r8*^*fl*/+^/Tg(*Krt14-Cre*) mice, which express (nearly) normal levels of NIPP1, were used as controls (CTRs). We compared the response of CTRs and SKOs with two-stage skin carcinogenesis, as induced by the topical administration of DMBA and/or TPA. Three different treatment regimens were adopted: (1) a single application of DMBA, (2) the administration of TPA twice a week for 20 weeks, and (3) a consecutive treatment with DMBA and TPA (Fig. 1a). No papillomas developed in the CTRs and SKOs after a treatment with DMBA or TPA alone (suppl. Fig. S1a, b), indicating that the deletion of NIPP1 does not initiate or promote tumor formation. With the combined treatment, the first papillomas in CTR mice appeared 6 weeks after the start of the TPA treatment (Fig. 1b–d; suppl. Fig. S1c, d). By 14 weeks, all CTR mice had developed 1–39 papillomas. In contrast, only one of six SKO mice had grown a (single) papilloma by this time, and this papilloma was small compared with most papillomas formed in CTR mice (suppl. Fig. S1e). These data demonstrated that NIPP1-deficient keratinocytes are strongly resistant to DMBA/TPA-induced skin carcinogenesis.

### Decreased mutagenic response to DMBA in SKOs

Metabolically activated DMBA forms covalent DNA adducts with adenine (A) that base pairs with A during DNA replication^18^. During the subsequent round of replication, the mutation is fixed as thymine (T) and is incorporated opposite to A. To examine whether the absence of NIPP1 hampers tumor initiation by DMBA, we quantified oncogenic A → T transversions in codon 61 of *Hras*^19^, which account for a major fraction of oncogenic mutations in DMBA-induced skin carcinoma^20^. For this purpose, genomic DNA was isolated from the skin of mice treated with TPA for 20 weeks or with DMBA (single application) plus TPA for either 5.5 weeks or 20 weeks. The oncogenic A → T transversion in codon 61 of *Hras* (HRAS^Q61L^) was massively enriched in tumors of CTR mice (Fig. 1e; suppl. Fig. S1f-h). To a lesser extent, this mutation was also present in tumor-free skin of CTR mice that had been treated with DMBA and TPA for either 5.5 weeks (no papillomas formed yet) or 20 weeks (papillomas formed). In contrast, the examined *Hras* mutation was barely detectable in the skin of CTRs treated with TPA alone and in SKOs treated with DMBA + TPA for 5.5/20 weeks. These data suggested that the deletion of NIPP1 severely impeded the mutagenic response to DMBA.

Next, we investigated whether the resistance of SKOs to papilloma formation was due to a skewed metabolism of DMBA. DMBA binds to the aryl hydrocarbon receptor (AHR), resulting in the upregulation of the Cytochrome P450 enzymes CYP1A1 and CYP1B1^21^. Together with epoxide hydrolase 1 (EPHX1), these P450 enzymes convert DMBA into a thiol epoxide that forms covalent DNA adducts^22^. We observed no differences in the (altered) expression of *Ahr*, *Cyp1a1*, *Cyp1b1*, *Ephx1*, and the AHR nuclear translocator (*Arnt*) between CTR and SKO mice following a treatment with DMBA for 0–24 h (suppl. Fig. S1i), hinting at a similar DMBA-biotransformation potential. Application of DMBA also provoked a similar apoptotic response in CTR and SKO mice, as indicated by similar expression levels of pro- and anti-apoptotic genes, and by similar numbers of cleaved Caspase-3-positive cells in CTR and SKO mice (suppl. Fig. S1j-l).

In the DMBA/TPA-induced skin carcinogenesis model, TPA induces inflammation and epidermal hyperplasia, providing a beneficial microenvironment for the survival and expansion of keratinocytes with oncogenic *Hras* mutations^16,23^. This prompted us to examine whether the absence of NIPP1 limits papilloma formation by reducing the hyperplastic response to TPA. However, the hyperproliferation phenotype of SKO epidermis was unaffected by a treatment with DMBA alone while a treatment with TPA increased epidermal thickness in both CTR and SKO mice (Fig. 1f–h). Strikingly, epidermal thickness in TPA-treated mice was 1.90 ± 0.22-fold more increased in the SKOs than in the CTRs (Fig. 1h), indicating that the ablation of NIPP1 even amplified the hyperplastic response to TPA. Overall, our findings suggested that the reduced sensitivity of the SKOs to induced skin carcinogenesis stemmed from a decreased conversion of DMBA-induced DNA adducts to overt mutations, despite an increased hyperplastic response to TPA.

### Increased DNA-damage response and DNA-repair capacity in SKOs

**Fig. 2:**
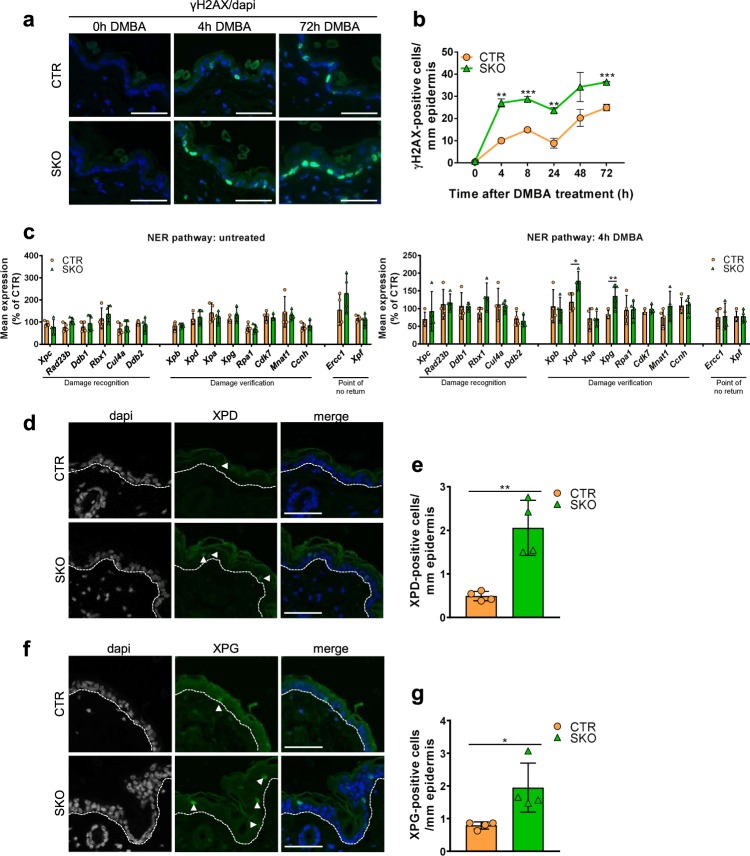
Enhanced DNA-damage response and repair in SKOs. **a** Immunostaining for γH2AX at the indicated time points after DMBA administration. Dapi was used for nuclear staining. Scale bars, 50 µm. **b** Quantification of γH2AX, as shown in panel a. The data are expressed as means ± SEM (*n* = 4). **c** qRT-PCR analysis of NER factors in the back skin of mice not treated (left panel) or treated for 4 h with DMBA (right panel). *Hprt* was used as a housekeeping gene for normalization. The data are expressed as means ± SD (*n* = 4). **d** Immunostaining for XPD in the back skin of mice treated for 24 h with DMBA. Arrows indicate positive stained cells. Dapi was used as a nuclear counterstain. Scale bars, 50 µm. **e** Quantification of the data in panel (**d**). The data are expressed as means ± SD (*n* = 4). **f** Immunostaining for XPG in the back skin mice treated for 24 h with DMBA. Arrows indicate positive stained cells. Dapi was used as a nuclear counterstain. Scale bars, 50 µm. **g** Quantification of the data in panel (**f**). The data are expressed as means ± SD (*n* = 4). *p < 0.05; **p < 0.01; ***p < 0.001 in unpaired Student’s *t*-test.

To obtain more insights in the reduced mutagenic response of the SKOs to DMBA, we examined the DNA-damage response at various time points after the administration of DMBA. The histone H2A variant H2AX is rapidly phosphorylated at S139 (γH2AX) when DNA is damaged, e.g., by DMBA administration^19,24^. Strikingly, the accumulation of γH2AX in DMBA-treated skin was about 2-fold more pronounced in the SKOs, as compared with that in the CTRs, hinting at an increased DNA-damage response (Fig. 2a, b). DMBA-induced DNA adducts are mainly detected and repaired through the nucleotide-excision-repair (NER) pathway, but can also be converted to DNA double-strand breaks^25^. Quantitative qRT-PCR analysis of all key regulators of the NER pathway did not reveal differences in transcript levels between non-treated CTRs and SKOs. However, following the administration of DMBA, the transcripts encoding the XPD helicase and the XPG endonuclease, which are required for DNA melting around the lesion and lesion excision, respectively, were increased in SKO mice (Fig. 2c). Immunostaining confirmed an increased number of XPD- and XPG-positive cells in the SKOs following DMBA treatment (Fig. 2d–g). Together, these data indicated that the accumulation of DMBA-induced mutations was reduced in *Ppp1r8*^*−/*−^ keratinocytes by an increased DNA-damage response as well as an upregulation of rate-limiting DNA-repair factors.

### Resistance of *Ppp1r8*^*−/*−^ livers to DEN-induced hepatocellular carcinoma

**Fig. 3:**
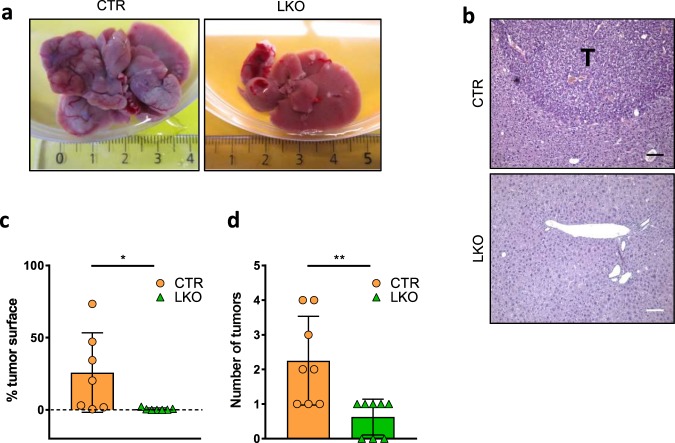
*Ppp1r8*^*−/*−^ livers are less sensitive to DEN-induced hepatocellular carcinoma. **a** Representative picture of CTR and LKO livers 10 months after a single intraperitoneal injection of DEN (25 mg/kg) in male mice of 14 days. **b** H&E stainings of the DEN-treated livers. Scale bar, 100 µm; T, tumor tissue. **c** The tumor surface area in DEN-treated livers was quantified on H&E-stained sections. The data are expressed as means ± SD (*n* = 7). **d** Number of tumors, as detected in on H&E sections (*n* = 8). All Data are expressed as means ± SD. *p < 0.05; **p < 0.01 (unpaired Student’s *t*-test).

Next, we asked whether the resistance of *Ppp1r8*^*−/*−^ cells to mutagenesis also applies to an unrelated mouse model of induced tumorigenesis. For that reason, we compared the response of liver control mice (CTRs; *Ppp1r8*^fl/+^/Tg(Alfp-Cre)) and NIPP1 liver knockout mice (LKOs; *Ppp1r8*^fl/−^/Tg(Alfp-Cre)) to chemically induced hepatocellular carcinoma (HCC). In the LKOs, the second floxed *Ppp1r8* allele was selectively disrupted in hepatoblast-derived epithelial cells, i.e., hepatocytes, cholangiocytes and their bipotential liver progenitor cells, by a *Cre* recombinase under control of the albumin promoter with enhancer elements from both the albumin and α-fetoprotein genes^14^. HCC was induced with a single intraperitoneal injection of the alkylating agent diethylnitrosamine (DEN). In accordance with published data^26–28^, numerous tumor nodules were macroscopically detected after 10 months in all CTR livers (Fig. 3a). H&E stainings showed that ≈25% of the liver sections of CTR mice consisted of tumor tissue (Fig. 3b, c). In contrast, few nodules appeared in the LKOs and these were generally much smaller (Fig. 3b, d).

### Increased expression of the DNA-alkylation repair enzyme MGMT in *Ppp1r8*^−/−^ cells

**Fig. 4:**
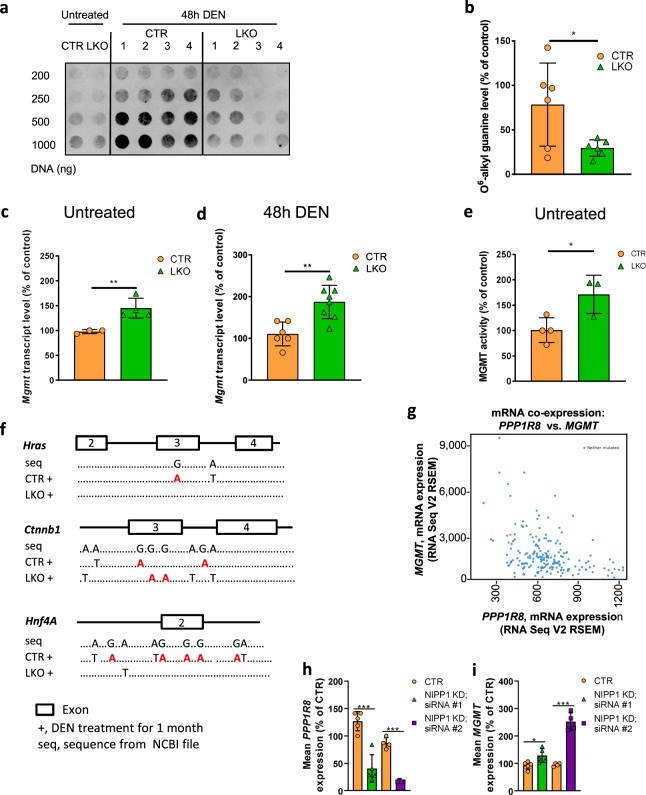
Increased expression of MGMT in liver cells upon depletion of NIPP1. **a** O^6^-alkyl guanine levels were measured by dot-blot assays on genomic DNA isolated from livers of male 14-days-old CTR and LKO mice that had been injected intraperitoneally with 25 mg/kg DEN 48 h before sacrifice (*n* = 4). Genomic liver DNA was loaded onto a nitrocellulose blotting membrane and probed with an antibody against O^6^-alkyl guanine (Squarix, EM2-3). **b** Quantification of the O^6^-alkyl guanine levels. *Hprt*, as determined by qPCR, was used for normalization. The data are expressed as means ± SD (*n* = 6). **c**, **d** *Mgmt* transcript levels were measured by qRT-PCR in the livers of untreated CTR and LKO mice (*n* ≥ 3) of two weeks (**c**) and 48 h after a DEN injection (25 mg/kg) in 14-days-old CTR and LKO mice (**d**; *n* ≥ 6). *Hprt* was used for normalization. The data are expressed as means ± SD. Total RNA was isolated from snap-frozen mouse livers using the GenElute^®^Mammalian Total RNA Miniprep kit (Sigma). **e** MGMT activity in liver extracts from untreated CTR and LKO mice of two weeks old. MGMT activity was measured according to manufacturer guidelines (MD0100, Sigma-Aldrich). The data are expressed as means ± SD (*n* ≥ 3). Immunoblotting with Tata-binding protein (TBP; Abcam, ab51841) was used for normalization. **f** Graphic display of mutations in the indicated gene fragments, 1 month after a single injection of DEN (25 mg/kg) in male CTR and LKO mice of 14 days. Proofreading PCR for *Hnf4a*, *Ctnnb1*, and *Hras* was performed on genomic liver DNA from 4 CTR and 4 LKO mice. PCR products were cloned into the pGEM^®^-T easy Vector System (Promega) and processed for sequencing. All observed mutations are indicated in the figure. **g** Negative correlation between *PPP1R8* and *MGMT* mRNA expression levels in human liver samples with hepatocellular carcinoma. The graph was derived from data generated by the TCGA Research Network (http://cancergenome.nih.gov/ and www.cbioportal.org). **h**, **i** HepG2 cells were treated during 72 h with control (Ctr) siRNA or two independent NIPP1 siRNAs (NIPP1 KD, siRNA#1 or siRNA#2). Knockdown efficiency and *MGMT* expression were analyzed by qRT-PCR. *HPRT* was used for normalization. All data are means ± SD (*n* ≥ 4). *p < 0.05; **p < 0.01; ***p < 0.001 (unpaired Student’s *t*-test).

A metabolite of DEN causes HCC because it ethylates guanine (G) at O^6^ or N^7^ and adenine (A) at O^3^^27^. The predominant mutagenic lesion caused by DEN is an ethylation of guanine at O^6^. O^6^-ethylguanine base pairs with thymine (T). Hence, if the alkylation of a guanine remains unrepaired it generates a G → A transition during the subsequent round of DNA replication. Using an antibody that is specific for alkylated O^6^-guanine, we found that LKO mice were protected from acute DEN-mediated guanine alkylation (Fig. 4a, b), consistent with their much reduced tumor burden 10 months later (Fig. 3). A reduced alkylation can be due to a deficient conversion of DEN into its alkylating metabolite and/or more efficient alkylation repair. Biotransformation of DEN involves its cytochrome P450-mediated hydroxylation, resulting in the release of acetaldehyde and a reactive ethyldiazonium ion^27^. The expression of the P450-encoding *Cyp* genes was not significantly affected by the deletion of NIPP1, except for a small increase in the expression of *Cyp1a2* (suppl. Fig. S2a), hinting at a normal biotransformation potential. Therefore, we explored whether the LKOs showed a more efficient ethylation repair. Alkylated O^6^-guanines are mainly repaired by the suicide enzyme O^6^-methylguanine-DNA methyltransferase (MGMT)^29^. qRT-PCR analysis showed that the *Mgmt* transcript level was increased by 50–100% in both untreated (Fig. 4c) and DEN-treated LKOs (Fig. 4d). The increased *Mgmt* transcript in LKOs was validated in the previously published RNA sequencing data^14^. The catalytic activity of MGMT was also significantly increased following the depletion of NIPP1 (Fig. 4e). The increased ethylation-repair potential of the LKOs was confirmed by sequencing of randomly selected gene fragments from three genes, which identified a 75% reduction in DEN-induced G → A conversions in the LKOs (Fig. 4f). Importantly, the number of another type of DEN-induced mutation (A → T transversions), which is not repaired by MGMT, was similar in CTRs and LKOs. This represents further evidence for a normal biotransformation of DEN in the LKOs. Collectively, these data indicated that DEN was equally efficient in the ethylation of DNA in CTR and LKO mice, but that O^6^-ethylated guanines were more efficiently repaired in the LKOs because of an increased expression of MGMT.

Interestingly, database mining also revealed a negative correlation between the transcript levels of *PPP1R8* and *MGMT* in human HCC (Fig. 4g), indicating that NIPP1 also opposes the expression of *MGMT* in human liver. Consistent with this notion, the knockdown of *PPP1R8* in the human HCC cell line HepG2 with two different siRNAs was associated with a significantly increased level of the *MGMT* transcript, albeit to a somewhat different extent (Fig. 4h, i). Immunoblotting analysis after the siRNA-mediated knockdown of NIPP1 in human HAP1 cells also revealed an increased MGMT level (Fig. S2b, c). Hence, the regulation of MGMT by NIPP1 is conserved in man.

## Discussion

Here we have demonstrated that the deletion of NIPP1 in epithelial cells of mouse liver or skin confers resistance to chemically induced mutagenesis and carcinogenesis. This finding was unexpected as the SKO and LKO models develop a spontaneous hyperproliferation phenotype, which is usually associated with an increased sensitivity to tumorigenesis^30,31^. Our data identify NIPP1 as a repressor of DNA repair, which is consistent with our previous finding that overexpression of NIPP1, as a fusion with PP1, causes a spontaneous accumulation of DNA damage^3^. Different types of DNA lesions evoke distinct DNA-repair mechanisms^25^. In the present study, we focused on DNA damage repaired through the MGMT or NER pathways. However, PP1-NIPP1 is also implicated in the repair of DNA double-strand breaks^3^, indicating that NIPP1 is a more general repressor of DNA repair. The resistance of the NIPP1 SKOs and LKOs to genotoxic stress correlated with an enlarged activation of the DNA-damage response as well as an increased expression of DNA-repair factors, two key determinants of the DNA-repair capacity.

The mechanism underlying the increased DNA-repair capacity of the NIPP1 SKOs and LKOs is currently unclear. We speculate that the loss of NIPP1 results in the hyperphosphorylation and activation of FHA ligands known to promote DNA-damage signaling, expression of DNA-repair proteins or DNA repair itself (see Introduction for references). However, since NIPP1 acts as both an inhibitor and activator of associated PP1^32,33^, it cannot be excluded that the deletion of NIPP1 actually reduces the phosphorylation of (some) FHA ligands. In addition, hitherto unidentified FHA ligands may be involved in the NIPP1-mediated response to DNA damage. Finally, we cannot rule out a more indirect mechanism. For example, the hair-follicle stem cells in the SKOs are less abundant and show a reduced stemness^15^. Since hair-follicle stem cells are the primary target for the induction of papillomas by DMBA/TPA^34^, it seems possible that the SKOs do not provide the stem-like state that is required to induce and propagate the oncogenic *Hras* mutation in codon 61.

The identification of NIPP1 as a general inhibitor of the DNA-repair capacity turns this protein into a potential target for therapeutic intervention. Patients with *Xeroderma pigmentosum* (XP) are extremely sensitive to UV-induced skin cancer, mostly because of an inactivating mutation of a key component of the NER pathway^35^. Our data suggest that the inhibition or removal of NIPP1 may offer resistance to UV-induced mutations for a subset of *Xeroderma pigmentosum* patients with hypomorphic mutations in NER pathway components. Conversely, induction of DNA damage through activation of NIPP1 is a possible new strategy for cancer therapy, as such or in combination with clinically used DNA-damaging treatments.

In conclusion, using two distinct mouse models for induced tumorigenesis, we have demonstrated that the loss of NIPP1 increases the DNA-repair capacity. This brings NIPP1 in the limelight as a novel target for cancer prevention or treatment therapies.

## Materials and methods

Detailed information on the animal models, chemically induced carcinogenesis models, Quantitative reverse transcriptase PCR, quantification of the genomic mutations and histological, immunohistochemical and statistical analyses is available in the supplementary section. Primers for genotyping and for q(RT)-PCR and antibodies are listed in supplementary Tables 1–3.

## Supplementary information

Supplemental Material
