# Supplementary material for: Enhanced DNA-repair capacity and resistance to chemically induced carcinogenesis upon deletion of the phosphatase regulator NIPP1
Source: Oncogenesis. 2020 Mar 2;9(3):30. doi: 10.1038/s41389-020-0214-3 (PMC7051951; doi:10.1038/s41389-020-0214-3)
Supplement: Supplementary file 1 — Supplemental Material [file 41389_2020_214_MOESM1_ESM.pdf]

Enhanced DNA-repair capacity and resistance to chemically induced carcinogenesis upon deletion of the phosphatase regulator NIPP1

Iris Verbinnen, Shannah Boens, Monica Ferreira, Kathelijne Szekér, Louise Van Wijk, Aleyde Van Eynde, and Mathieu Bollen

Laboratory of Biosignaling & Therapeutics, KU Leuven Department of Cellular and Molecular Medicine, University of Leuven, Belgium

Supplementary information

- Supplementary figures S1 and S2.
- Legends to the supplementary figures.
- Materials and methods.
- Supplementary table S1: primers for genotyping.
- Supplementary table S2: qRT-PCR primers.
- Supplementary table S3: antibodies.
- Supplementary references.

Supplemental Figure 1

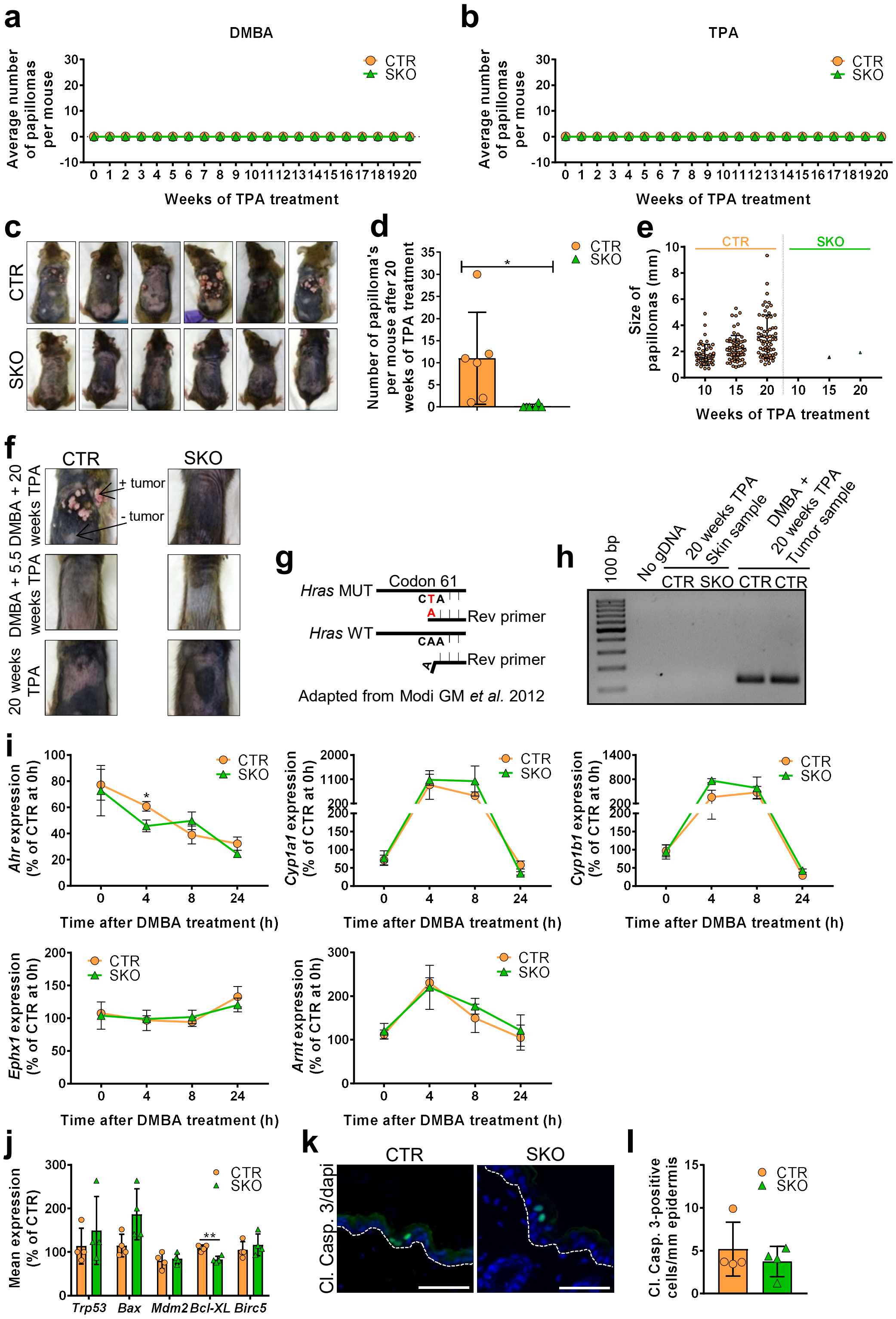

Supplemental Figure 2

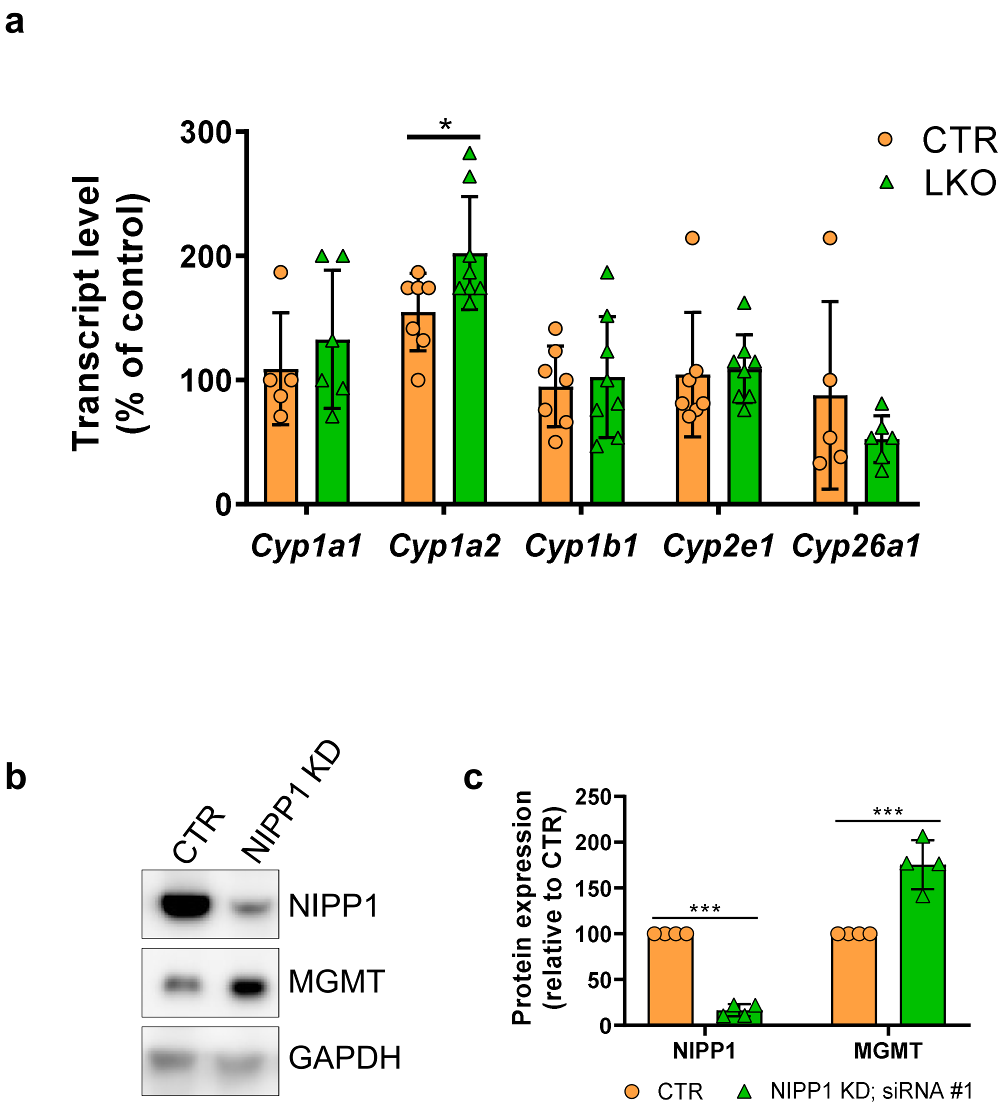

LEGENDS TO THE SUPPLEMENTARY FIGURES

Suppl. fig. 1. The response of SKO mice to DMBA and/or TPA treatment. **a** Papilloma formation in mice treated with only DMBA (n=6) **b** Papilloma formation in mice treated with only TPA (5 CTR and 7 SKO) **c** Pictures of all CTR and SKO mice treated with DMBA + 20 weeks TPA (n=6). **d** The average number of papillomas per mouse at the end of the treatment with DMBA + TPA (n=6) *, $p<0.05$ (unpaired Student's t-test). **e** Papilloma size during the DMBA + TPA treatment. All papillomas (Fig. S1c) were measured using a caliper. The individual data points are shown as well as the means ± SD (black lines). **f** *Hras* mutation screening was performed in three different groups: DMBA + 20 weeks TPA (tumors, tumor-free skin), DMBA + 5.5 weeks TPA, and DMBA + 20 weeks TPA. **g** Scheme of the method used for the detection of activating A→T transversions in codon 61 of the *Hras* gene. **h** Mutation-specific primers specifically recognized the A→T transversion in codon 61 of the *Hras* gene. As a negative control, genomic DNA was isolated from skin of mice that were treated with only TPA for 20 weeks. **i** Response of the indicated transcripts to single topical application of DMBA. *Hprt* was used as a housekeeping gene for normalization. For measuring *Cyp1a1* and *Cyp1b1* expression, *Cyp1a1*_set1 and *Cyp1b1*_set1 primers were used respectively (**Table S2**). Data are represented as means ± SEM (n=4). *, $p<0.05$; **, $p<0.01$ (unpaired Student's t-test). **j** Expression levels of the indicated pro- and anti-apoptotic genes in back skin samples 24h after DMBA treatment. *Hprt* was used as a housekeeping gene for normalization. Data are shown as means ± SD (n=4). **, $p<0.01$ (unpaired Student's t-test). **k** Immunostaining for cleaved caspase 3 in back skin sections 24h after DMBA. Dapi was used for nuclear staining. Scale bars, 50 µm. **l** Quantification of the data shown in panel k. The results are expressed as means ± SD (n=4) (unpaired Student's t-test).

Suppl fig. S2 a Hepatic expression of the DEN metabolizing *Cyp* genes after NIPP1 deletion. Transcript levels of *Cyp1a1*, *Cyp1a2*, *Cyp1b1*, *Cyp2e1* and *Cyp26a1* were measured by qRT-PCR in mouse livers 48h after a single injection of DEN (25mg/kg) in male mice of 14 days. *Hprt* was used for normalization. For measuring *Cyp1a1* and *Cyp1b1* expression, *Cyp1a1*_set2 and *Cyp1b1*_set2 primers were used respectively (**Table S2**). Data are represented as means ± SD (n≥5). *, $p<0.05$ (unpaired Student's t-test). **b** The level of NIPP1 and MGMT in cell lysates of HAP1 cells, which were treated with control (Ctr) or NIPP1 siRNA#1, was visualized by immunoblotting. GAPDH served as a loading control. **c** Quantification of the immunoblot analyses of 4 independent siRNA-mediated knockdowns in

HAP1 cells, as shown in S2b. The results are expressed as means ± SD (n=4) (unpaired Student's t-test).

Materials and methods

Animal models and experimental design

Mice were housed in a specific pathogen-free animal facility under standard 12h-light/dark cycles with water and chow *ad libitum*. Transgenic (Tg) *Krt14-Cre* mice and Tg(*Alfp-Cre*) mice were kind gifts from Dr. Walter Birchmeier (Max-Delbrueck-Center for Molecular Medicine, Berlin, Germany) and Dr. Frédéric Lemaigre (UCL, Belgium), respectively. The generation of *Ppp1r8*$^{fl/fl}$ mice is described in (1). DNA used for genotyping was extracted from ear biopsies. PCR primers are described in **Table S1** and the PCR conditions are available on request. All animals were anaesthetized and sacrificed according to institutional guidelines. Skin and liver sections were either directly frozen in liquid nitrogen or fixed in PBS containing 4% formaldehyde. All experimental protocols were in accordance with and approved by the Guide of Care of Experimental Animals of the KU Leuven Ethical Committee (license numbers: 036/2013 and 053/2018).

Chemical carcinogenesis

For chemical-induced carcinogenesis models in skin and liver, no statistical methods were used to pre-determine samples size or for randomization. Littermates were randomly allocated to the experimental group according to their genotype. For the chemical-induced skin carcinogenesis model, female mice of 7-8 weeks of age were shaved using a fine electric shaver (B|Braun, Aesculap®). Two days after hair removal, a single initiation dose of 50 µg DMBA (Acros Organics) in 200 µl acetone was applied to the shaved area. Starting two weeks after DMBA application, the DMBA-initiated area was treated twice weekly with 6 µg of TPA (Acros Organics) in 200 µl acetone. Mice weight and the appearance of papillomas were monitored and recorded every week. Papillomas with a diameter bigger than 1 mm were counted. The number and size of papillomas per mouse were examined for up to 20 weeks. Also, two control groups, encompassing mice that either received DMBA or TPA, were set up. In the control group of only TPA treatment, one mouse died during the experiment, therefore only data from 5 mice were analysed in this group. No other mice were excluded. For short-term DMBA applications, mice were treated with 50 µg DMBA in 200 µl acetone and sacrificed after 4h, 8h, 24h, 48h or 72h. For the DEN-induced hepatocellular carcinoma

model, male mice (n=8) of 14 days were treated with a single intraperitoneal injection (25 mg/kg) of *N*-Nitrosodiethylamine (Sigma-Aldrich). CTR and LKO livers were collected 10 months after injection and examined for the number of tumors and tumor surface area, as detected on H&E sections.

Quantitative Reverse Transcriptase PCR

Total RNA was isolated from HepG2 cells or from snap-frozen mouse tissues (total skin or liver), stored at -80°C, using the Genelute Mammalian Total RNA Miniprep kit (Sigma-Aldrich). Complementary DNA (cDNA) was synthesized from 2 µg of total RNA using oligo dT primers (Sigma-Aldrich), RevertAid Premium Reverse Transcriptase and RiboLock RNase inhibitor enzymes (Fermentas). 1.2% of the cDNA was PCR-amplified in duplicate using SYBR Green qPCR Mix (Invitrogen) and a Rotorgene detection system (Corbett Research) (1). To determine the relative amount of target in different samples, all values were normalized to the housekeeping gene *Hprt* (Hypoxanthine-guanine phosphoribosyltransferase). All used qRT-PCR primers can be found in **Table S2**.

Quantification of genomic mutations

To determine the genomic *Hras* mutations in the DMBA-induced SKO, full thickness murine dorsal skin was removed and genomic DNA (gDNA) was isolated by lysing skin samples for minimum 3 hours in TNES buffer containing 100 mM Tris-HCl at pH 8.8, 200 mM NaCl, 5 mM EDTA and 0.2% SDS supplemented with 0.1 mg/ml proteinase K. The lysates were centrifuged for 10 minutes at 12 000 *g* and DNA was precipitated with isopropanol. Finally, gDNA was dissolved in buffer containing 10 mM Tris-HCl at pH 7.5 and 1 mM EDTA. The purity and concentration of gDNA was analyzed with NanoDrop (Thermo Fisher Scientific). To quantify the number of DMBA-induced codon-61 *Hras* CAA → CTA mutations, qPCR with mutant-specific primers was performed on isolated gDNA as previously described (2). Obtained Ct (Cycle threshold) values were normalized against genomic *Actb*. Used primers can be found in **Table S2**. For the quantification of DEN-induced mutations in LKOs, proofreading PCR for *Hnf4a*, *Ctnnb1* and *Hras* was performed on liver gDNA of male 14 days-old CTR and LKO mice, 1 month after a single injection with DEN (25 mg/kg). PCR products were cloned into the pGEM®-T easy Vector System (Promega), and processed for sequencing. Eight clones of each gene were sequenced from 4 CTR and 4 LKO mice, leading to a total of 192 sequences which were further analyzed for mutations.

Histological and immunohistochemical analysis

Skin and liver tissues were immediately fixed in PBS containing 4% formaldehyde, and embedded in paraffin at the Histology & Imaging facility (Vesalius Research Center, KU Leuven). Skin and liver sections of 5 µm were stained with hematoxylin and eosin (H&E). The antibodies used for immunohistochemistry are described in **Table S3**. Microscopy was carried out using a Leica DMI 4000B microscope or Leica DMBL microscope and images were processed with Leica MM AF or Leica IM50 Image Manager respectively, ImageJ (National Institute of Health).

Biochemical procedures

O^6-alkyl guanine levels were measured by dot-blot assays on genomic DNA isolated from livers of male 14-days old LCTR and LKO mice that had been injected intraperitonially with DEN (25mg/kg) 48h before sacrifice. Genomic liver DNA was mixed with 0.4 M NaOH and 10 mM EDTA, and denatured for 10 min at 100°C. Samples were cooled on ice, neutralized with 2 M ammonium acetate at pH 7.0, loaded onto a nitrocellulose blotting membrane and probed with an antibody against O^6-alkyl guanine. *Hprt* levels, as determined by qPCR, were used for normalization. MGMT activity in liver extracts from untreated LCTR and LKO mice of two weeks old was measured according to manufacturer guidelines (MD0100, Sigma-Aldrich). Immunoblotting with TBP was used for normalization. Used antibodies can be found in **Table S3.** HAP1 cells, which were treated with control or NIPP1 siRNAs (Fig. S2b), were harvested in cold PBS and lysed in modified RIPA buffer containing 50 mM Tris-HCl at pH 7.4, 1% Triton-X100, 0.2% sodium deoxychlorate, 0.2% sodium dodecyl sulfate, 1 mM EDTA and 0.3 M NaCl. The lysis buffer was supplemented with 20 mM NaF, 5 µM leupeptin, 0.5 mM phenyl methanesulfonyl fluoride, 0.5 mM benzamidine and 1 mM orthovanadate. The lysates were incubated on ice for 20 min, centrifuged at 3,800 *g* for 10 minutes at 4°C. The supernatants were retained and processed for SDS-PAGE and immunoblotting. Proteins were blotted on polyvinylidene fluoride membranes (GE Healthcare) and probed with various antibodies (**Table S3**). Immunoblots were visualized using ECL reagent (Perkin Elmer, Life Sciences) in the ImageQuant LAS4000 imaging system (GE Healthcare) and were quantified using ImageQuant TL Software (GE Healthcare).

Cell culture and siRNA-mediated knockdown

HepG2 cells were a kind gift from J. Goris (Belgium) and tested negative for mycoplasma, while HAP1 cells (parental C631) were obtained from Horizon Genomics GmBH, Austria.

HepG2 and HAP1 cells were cultured in Dulbecco's modified Eagle's medium (DMEM), supplemented with 10% fetal bovine serum, 100 U/ml penicillin and 100 µg/ml streptomycin and 2 mM L-glutamine. Cells were treated during 72h with duplexes of siRNA targeted against human NIPP1 (siRNA#1 sequence: 5'-GGAACCUCACAAGCCUCAGCAAAUU-3'; siRNA#2 sequence: CACUUGUCUACCACAAGCAUCUGAA using Lipofectamine®RNAiMAX reagent (Invitrogen). Used siRNAs were purchased from Invitrogen.

Data and statistical analysis

No statistical methods were used to predetermine samples size and the investigators were not blinded to the group allocation during the experiments. The results are expressed as means ± standard deviations (SD) or as means ± standard error of the mean (SEM) and were analyzed with the two-sided unpaired Student t-test. Tumor incidence was analyzed using the Log-rank test. We assumed that the variance between the groups are similar because the groups consist mostly of littermates. All data that are shown as percentage (%) of CTR, are expressed as % of one randomly chosen CTR sample. Figures 1c-e, 1g-h, 2b-c, 2e, 2g, 3c-d, 4b-e, 4h, S1a-b, S1d-e, S1i-j, S1l and S2 were generated using the GraphPad Prism version 7.04 software (GraphPad Software, Inc.). Figure 4g was derived from data generated by the TCGA Research Network (http://cancergenome.nih.gov/ and www.cbioportal.org).

SUPPLEMENTARY TABLE S1: primers for genotyping.

Target	Sense Primer	Antisense Primer
Tg(K14-Cre)	CTTGCGAACCTCATCACTCG	AGGGATCTGATCGGGAGTTG
Tg(Alfp-Cre)	GCAAACATACGCAAGGGATT	CCTGATCCTGGCAATTTCGGC
Floxed *Ppp1r8* gene<br>- LoxP1<br>- LoxP2	CCACCCTCTCCTTTACTTTGTCTTC<br>CTTACAAGGAGTGGTATTCGA	GGAGAGGAGTAATGAGGAGTTGTG<br>ACTGTCTAGCAGGGCATAGTGTTG
Ppp1r8 null gene	CCTCAGCAGATAGCCCACGG	CGCATCGCCTTCTACGCCTTCTTGAC

SUPPLEMENTARY TABLE S2: qRT-PCR primers.

Gene	Sense Primer	Antisense Primer	Application
Actin	ATCAAGATCATTGCTCCTCCTGAG	CTGCTTGCTGATCCACATCTG	gDNA
Ahr	AGGACCAAACACAAGCTAGA	TGGAGATCTCGTACAACACA	cDNA
ArntI	CTACAAGCCAACATTTCTATCAGATGA	GGTCACATCCTACGACAAACAAAA	cDNA
Bax	CTCACGGAGGAAGTCCAGTGT	CATGTTTGCTGATGGCAACTT	cDNA
Bcl-xl	TGGTCGACTTTCTCTCCTACA	CCCTCTCTGCTTCAGTTTCTTC	cDNA
Birc5	TCTGGCAGCTGTACCTCAAG	ATCAGGCTCGTTCTCGGTAG	cDNA
Ccnh	GTAATGGAATATCACCCTCGGATAA	CTCTCCTGTCCAAGAGGACT	cDNA
Cdk7	TAGGATGTATGGTGTGGGAGTA	AAGATCTGAATCTCCAGGCAAA	cDNA
Cul4a	ACACATGCTGGCAAGATCA	CATGGAATTCTGGAGGACGTAG	cDNA
*Cyp1a1*_set1	CCGTTACCTGCCTAACTCTTC	GCCCTTCTCAAATGTCCTGTA	cDNA
*Cyp1a1*_set2	CTCTTTGGAGCTGGGTTTGACAC	AGGGTTGGTTACCAGGTACATGAG	cDNA
Cyp1a2	TCCTGGAGATCTACCGATACAC	TGACCTGCCACTGGTTTATG	cDNA
*Cyp1b1*_set1	TGGCTGCTCATCCTCTTTAC	GTTGGGCTGGTCACTCAT	cDNA
*Cyp1b1*_set2	GGATGTGCCTGCCACTATTA	CACAACCTGGTCCAACTCA	cDNA
Cyp26a1	AGAGCAATCAAGACAACAAGTTAG	ATCGCAGGGTCTCCTTAAT	cDNA
Cyp2e1	GGTGCTACTGAACCACAAGAA	TCACGGAGGATACTTAGGGAAA	cDNA
Ddb1	GGACAGGAATCAATCACCTATCA	CATTAGGATCCACCCGGTTATG	cDNA
Ddb2	AACCAGGCTGCAGGATTT	TCTCCTGTAACCACCACTCT	cDNA
Ephx1	CAGAGGCATCCAGCAAGAAA	CGCCGCCTTGAATGTAGAA	cDNA
Ercc1	CAACCTCCATCCAGACTACATC	CACATCTTAGCCAGCTCCTT	cDNA
Hprt	CTGGTGAAAAGGACCTCTCG	TGAAGTACATTATAGTCAAGGGCA	cDNA
*HPRT**	TGACACTGGCAAAACAATGCA	GGTCCTTTTCACCAGCAAGCT	cDNA
Hras MUT	CTAAGCCGTGTTGTTTTGC	CATGGCACTATACTCTTCT	gDNA

	AGGAC	A	
Hras WT	CTAAGCCGTGTTGTTTTGCAGGAC	CATGGCACTATACTCTTCTT	gDNA
Mdm2	GTCTATCGGGTCACAGTCTATCA	TTTATCTTTCCCCTTATCGTCTG	cDNA
Mgmt	CATGGGATACGGTTGCTCAG	TGCGGGTTCACGGAAATAG	cDNA
*MGMT**	GTGATTTCTTACCAGCAATTAGCA	CTGCTGCAGACCACTCTGTG	cDNA
Mnat1	TGAAGATCCCACTGTTGATAAGG	CGATTTCCTCCACTTCTTCCA	cDNA
Ppp1r8	AGCGCTGGTGTACCACAAACA	TTGTGAGGTTCCAGCCGAATGT	cDNA
*PPP1R8**	TACACACGGCACTTTCTTGG	CTTGTTGTGGGCAGTGTTGA	cDNA
Rad23b	GGGCTATGAACGAGAACAAGTA	CCACAGCCTGACTTTCTCTATC	cDNA
Rbx1	CCATCTGCAGGAACCACATTA	CCATGCAACCGTACACTCTT	cDNA
Rpa1	ATCCGCACCTGGAGTAATTC	CACTTGCTCATTGAAAGCAGTAG	cDNA
Trp53	AACCGCCGACCTATCCTTAC	CTTCTGTACGGCGGTCTCTC	cDNA
Xpa	GGACTCGTACCTTATGAACCAC	CTCTTGCTTCGCTTCTGTCT	cDNA
Xpb	CGCTCAGGAGTCATTGTTCT	CCACATCTTAAACTGGGCTTTC	cDNA
Xpc	GGCGGTGGAGATTGAAATTG	CTCCGCAGGTATGTCTCAAA	cDNA
Xpd	GCTCACACTCCTTGCTAACT	CGTCAAAGGGCTCAATGATAATG	cDNA
Xpf	TGTCACGTGGAAAGAGTGATG	GGCTTGTGTTGTTCCAAGAATG	cDNA
Xpg	CCAGTCTCACTCAGGTTCAAAG	GATCCATTCTTGCTTGCCATTG	cDNA

cDNA, complementary DNA; gDNA, genomic DNA

*, qPCR primer sequences used for HepG2 (human) transcripts.

SUPPLEMENTARY TABLE S3: antibodies.

Target protein	Company (Cat. no.)	Application
Cleaved Caspase 3	Cell Signaling Technology (9664)	IHC
γH2AX	Merck (05-636)	IHC
XPD	GeneTex (GTX105357)	IHC
XPG	Biorbyt (orb256523)	IHC
O^6-alkyl guanine	Squarix (EM2-3)	Dot-blot assay
TBP	Abcam (ab51841)	WB
MGMT	Millipore (MAB16200)	WB
NIPP1	Sigma-Aldrich (HPA027452)	WB
GAPDH	Cell Signaling Technology (2118))	WB
Alexa fluor 488 goat anti-rabbit IgG (H+L)	Thermo fisher Sci. (A11008)	IHC
Alexa fluor 488 donkey anti-mouse IgG (H+L)	Thermo fisher Sci. (A21202)	IHC
Alexa fluor 568 goat anti-mouse IgG (H+L)	Thermo fisher Sci. (A11004)	IHC
Swine anti-rabbit HRP	Dako (P0217)	IHC, WB
Rabbit anti-mouse HRP	Dako (P0260)	IHC, WB

IHC: immunohistochemistry; WB: western blot

SUPPLEMENTARY REFERENCES

1. Boens S, et al. Brief Report: The Deletion of the Phosphatase Regulator NIPP1 Causes Progenitor Cell Expansion in the Adult Liver. Stem Cells. 2016;34(8):2256–62.
2. Modi BG, et al. Langerhans cells facilitate epithelial DNA damage and squamous cell carcinoma. Science. 2012;335(6064):104–8.
